# Translation of a Chapter of the “Institutes of Medicine” of Heurnius, from the Latin

**Published:** 1846-10

**Authors:** John G. Affleck

**Affiliations:** Somerset, Ohio


Art. III.—*Translation of a Chapter of the "Institutes of Medicine" of Heurnius*, from the Latin. By Dr. John G. Affleck, of Somerset, Ohio.

Chance having, some years ago, thrown in my possession a very rare work, entitled the Institutes of Medicine, by J. Heurnius, printed at Leyden, in the year 1592, I have supposed that a translation of one of its chapters into English might be acceptable to the readers of the Western Journal, affording as it does a faint glimpse of the state of the medical art at the time it was written. I have been the more induced to lay this translation before those who peruse the Journal, from the circumstance, that as Dr. Coxe, of Philadelphia, proposes to translate the works of Hippocrates and Galen, and as Heurnius seems to be only an echo of those celebrated men, an insight into the secrets of those masters, so far as this scrap goes, may induce some to further the investigation, and may stimulate the profession to give encouragement to the Doctor's enterprize. It may not excite our wonder that Hippocrates, and Galen, and Avicenna, should have held the doctrines advocated in the following chapter, but that Heurnius, who wrote only a little over two hundred years ago, should retail the same absurdities, may well cause a smile.

The Institutes of Medicine were, in their day and generation, good authority, for we find one contemporary calling Heurnius "*Grande decus columenque rerum;*" another, "the most illustrious and the most learned;" and another, "the New Hippocrates."

He was born in Utrecht in 1543; studied at Louvain and Paris, and at Pavia took the degree of M. D. in 1571. In 1581 he accepted the medical professorship in the University of Leyden, and continued to discharge the duties for twenty years, during which time he had an extensive private practice. He was often consulted by William the First, Prince of Orange. In 1592 his "*Institutiones Medicinæ*" were published.

The Institutes consist of twelve books, of which the first treats of First Principles—the 2d of Temperaments—the 3d of Faculties—the 4th of Humors—the 5th of the procreation of man—the 6th of the difference of diseases—the 7th of the cause of disease—the 8th of the symptoms of disease—the 9th of the urine—the 10th of the pulse and respiration—the 11th of prognosis, and the 12th of Therapeutics or the method of healing. To which is added an essay on the mode of studying medicine.

From the following chapter of Heurnius, on the "procreation of man," it will be seen that in the time of Hippocrates, Galen and Avicenna, the signs of pregnancy were nearly the same as in our own. It is true we have made an accession to our list of several new ones, although few of them are of much value, in a practical point of view. Of the signs lately introduced, *ballottement* and *auscultation* have become fixed facts, and of immense value. *Jacquemin's test*, in this country, is of no practical worth, if true. *Gravidine* is yet sub judice, and *kiesteine*, although it has lately come into notice, under the auspices of Nauche, existed as a test of pregnancy, according to Heurnius, in the days of Avicenna. The original passage in Heurnius is, "Avicenna meminit urinæ ineunte pregnatione subalbidæ, tenuis, cui grana quædem innatare dicit." Would it not be worth the attention of some of our savans to examine the writings of Avicenna, and ascertain how much credit the late discoverer is entitled to? As a test, although creeping gradually into favor, it cannot be recognized as of primary importance.

The strange conceits about fruitfulness, twins, &c., will doubtless be estimated by the reader at their intrinsic value. In translating the chapter, I have followed the idiom of the author as nearly as possible, and have also added a few more extracts from different parts of the work.

Book v., Chapter v.—*Of the Signs of Conception—Of the Marks of Fecundity—Whether the Woman carries Male or Female, and whether Twins.*—About to treat of the signs of conception, let us first lay down the marks of fecundity—then the evidences of conception, and in the third place the signs which indicate whether a male or female fœtus is carried.

Hippocrates, pointing out the signs of fecundity, says, it is necessary to put a lotion upon the head, and dry it with lint free of all smell. In the evening, put galbanum upon the woman's bosom, and in the morning, while she is yet fasting, make warm the top of the head. If the galbanum sends up an odor, she will be fruitful, otherwise, not. So also in the aphorisms, he orders the woman to be perfumed with storax, frankincense and myrrh, and if the smell strikes against the nostrils and mouth, it argues fecundity. In addition to these signs, Avicenna says that barley should first be steeped in the urine of the man, then of the woman, and if it germinates when committed to the earth, it denotes fruitfulness. He also recommends sulphur to be sprinkled upon the urine, and if in time it breeds worms, it is an argument for fecundity. Those of a warm temperament, and in whom the right testicle swells, mostly beget boys.

Whether a woman hath conceived you may determine by the semen not escaping, and from the mouth of the womb being shut. But obstetricians are admonished, that this shutting up of the uterus ought to be soft, without hardness like an unnatural swelling. Besides, the menses stop, without fever and chills; but with nausea or pica, that is she will have a longing for strange things, occasioned by bad juices originating in the stomach. But you will say, Galen affirms in the 5th book, aphorism 60, that a woman sometimes is pregnant while the menses flow. Thus we say, and by this very flux the matter can be proved, for there are veins in the neck of the uterus which sometimes pour out abundantly the dregs which are in the veins of the womb, and which are collected between the second and third membranes; and thus in flowing it becomes of a pale color. But in what manner, therefore, if the blood thus flows, will we know if conception has taken place? We will know this from the copiousness and quality of the blood, which differs from that which is emitted from the unimpregnated uterus. From the impregnated woman it flows scant and corrupted—of an unsightly color, and useful neither for the fœtus nor mother. The blood in the impregnated woman goes out only through the veins, to the neck of the uterus, and not also through the veins of the body of the uterus. Besides, an evidence is given that the woman hath conceived, from the motion which is felt about the region of the uterus. But this happens also from flatus; true it is, but then the breast with the nipples will not be swollen; when the belly swells the breasts will also; this is almost in the last months. However, it may seem contrary to nature, that the breasts are sure to swell in the last months. Why not rather in the first months? for at this period a great deal of the excrementitious juices flows through the mammæ. We reply: the excrement is sent into the veins of the uterus, and finally they being expanded and filled, the blood is forced in another direction. The swelling of the abdomen, which presses upon the veins already filled, also favors it. But one will say, in what manner in the first months will the uterus membranous and thin, receive so much excrement; for by tension the uterus is rendered very thin. Believe both Aurantius and myself, who have seen it in dissection, that the substance of the uterus in the impregnated woman does not become thin, as was formerly supposed, but grows thicker. The uterus eventually is changed from a membranous substance to one unlike any thing else in the whole body. It is a porous body like a sponge, and divisible into many layers and pervious like pumice, except the external tunic, which is connected with the peritoneum. When the fœtus has almost arrived at maturity the uterus nearly equals the thickness of two fingers, by which it becomes suitable for the excrement. At last the whole uterus is filled, and pressure in the last months being made upon the abdomen, by the swelling which compresses the full veins of the uterus, the blood takes another direction, and is carried rather to the spongy void of the glands, on account of the genuine sympathy which exists between the uterus and breasts, than to it; thither, therefore, the blood directs its course. Besides, in pregnancy the eyes become hollow and pale, for the reason that they are deprived of animal spirits, which, as they are made from vital spirits, are not carried equally to the brain, but rather to the uterus. Avicenna makes mention, in pregnancy, of the urine being pale and thin, upon which certain grains float. But this is a vain idea, for the urine passes neither through the uterus nor the veins of the uterus.

Now I will give a few signs how we may know whether the mother carries a male or female in the uterus. Hippocrates, in his book "De natura pueri et de formatione fœtus," says if a male fœtus be carried, the right breast will swell first, and the right eye will be more lively. The reason is that the male is for the most part conceived in the right part of the uterus, and the woman will be of a more lively color; for when the male draws nourishment he infuses the masculine strength throughout the whole body, not otherwise than the testes do. But a woman ought not to be compared with others, but only with herself; that is, whether she is more sightly in the impregnated or unimpregnated state, and whether she enjoys herself the same as formerly, by reason of her way of living. For if she hath entered upon a better life after she hath conceived, although she may be pregnant with a female child, her color will be better than formerly. So if she carries a male and lives indifferently, her former color will be destroyed. But amongst all the signs, the motion of the infant is the most certain, for the male moves itself more vehemently. The female sometimes does the same, and on this account Avicenna prudently adds, that the male does it before the fourth month; for it begins to be moved in the third month. If it is a male, the woman will perceive the motion in the right side of the uterus, and the pulse of the right arm will beat more forcibly—the contrary, if a female. Avicenna adds that the right breast not only swells, but even pours out milk. He says you may pour the milk upon a glass, and expose it to the sun, and if it concretes like a spangle, she carries a male—if it is scattered, a female; for while a male is carried, heat increases; whence the milk becomes thicker. Hippocrates adds, if milk is spread upon lettuce, and it flows off, it denotes that the woman carries a female; and he says if the milk is mixed up with flour, and the mass put upon the fire, it indicates that it is a male if it can be toasted; but if dissolved, it is a presage that she carries a female.

But can it be known before, and in parturition, if the woman is big with twins. Before, if the tumor of the abdomen is large and she is of an unusual size. Avicenna asserts it is known in parturition, by the umbilical* cord of the first fœtus forcing its way out. If it has knot or wrinkle, the idea is that another fœtus remains in the uterus.

*I am not certain whether the umbilicus or umbilical cord is here meant.

*Extract from the Essay on the Mode of Studying Medicine.*—Hippocrates has always been esteemed the true exponent of the healing art. His writings are as oracles, and not the words of human speech. All antiquity cherishes him, as the author and director of all discipline. Celsus says he was a man renowned in science and richness of expression, and Macrobius says he neither knew how to deceive or to be deceived. In him there was a careful propriety of words—a concise accuracy of sentences—a venerated antiquity of speech—a commendable dignity of artifice. In his writings nothing is superfluous—nothing useless; but all expressed with beautiful brevity and significance; and the richness and amplitude of his facts far surpasses the number of words. These are natural and peculiar to Hippocrates. His benignity was so great that he knew nothing which he wished others not to know, and so great were his genius and wisdom that no one following him knew what he himself was ignorant of. In him you may find all the rudiments of medicine.

His most faithful commentator is Galen, who, as some say, lived one hundred and forty years. He, endowed with all philosophy and suitable philology, speaks every where in a pure, elegant, simple and never affected speech, not without a great store of words and sentences. He never offends the reader by satiety, but every where smooths the way of him desirous of progress, by rhetorical artifice. A man made for eloquence. In him you will find rare and select philosophical precepts, and more important than in any other writer. He obtrudes nothing upon the reader, which he does not demonstrate by strong reasons, and gives refutation to his opponent. He has very beautiful morals and subdued passions. He inveighs particularly against ambition and avarice, and treats learnedly and elegantly upon any subject. To no one, for the cultivation of his mind, are his words useless.

*Extract from Book vii., on the Cause of Diseases.*—According to Hippocrates, no cause predisposes more to disease, than the retention, or too great effusion of the secretions; such as mucus, bile, excremens, urine, the menses and hæmorrhoids. Retained flatus is fruitful of disease, as you may understand by the edict of the emperor Claudian, who, as Suetonius tells, gave the privilege by a proclamation, of belching and breaking wind at entertainments, when it was ascertained that by retaining them through shame the health would be in jeopardy.

Venery also may be reckoned amongst the excretions. Galen, in his books upon the medical art, writes that Epicurus, the greatest philosopher, esteemed the use of venery to be unhealthy; but not without reason, for he held in contempt corporeal pleasures. For he was a temperate man, as Diogenes Laertius writes, and a good man—pious and a worshiper of God and friends. However, according to the opinion of Pythagoras, coition ought to be used by those who wish to become more reduced. Immoderate venery brings on infirmity of strength and decrepid youth, which, according to the opinion of Cato, as Pliny relates, indicates an early death; because by copulation the spirit is drawn from the brain, and as the benignant humor is suddenly poured out from the solid parts, they are defrauded of their peculiar properties, and native humidity. Truly nothing equally enfeebles the brain, says Cælius Aurelianus, makes weak the body, and disturbs the substance of the soul, like constant venery. Hence, as Pliny tells, Democritus condemned all coition. Venery is bad while the menses flow; and the conception is unhappy when the system is imbued with foul disease, as well as when it takes place with polluted blood; hence elephantiasis and lepra. Copulation becomes fruitful in those in whom the menses have just flowed, and to those who are, as if it were, hidden. Whence the ancients said that, under the moon, Venus, who dances before, leads the band Venery is hurtful in tender age; whence Cæsar, in the sixth book of the Gallic war, writes, it is esteemed base to have connection with a woman before the twentieth year.

